# Conceptualizing the effects of the COVID-19 pandemic on people with opioid use disorder: an application of the social ecological model

**DOI:** 10.1186/s13722-020-00210-w

**Published:** 2021-01-07

**Authors:** Ethan Cowan, Maria R. Khan, Siri Shastry, E. Jennifer Edelman

**Affiliations:** 1 Department of Emergency Medicine, Icahn School of Medicine At Mount Sinai, 1 Gustave L. Levy Place, New York, NY 10029 USA; 2 Department of Population Health, NYU Grossman School of Medicine, 227 East 30th Street, Sixth Floor, 614, New York, NY 10016 USA; 3 Department of Internal Medicine, Yale School of Medicine, ES Harkness Memorial Hall, Suite 401, 367 Harkness Street, New Haven, CT 06510 USA; 4 Mount Sinai Beth Israel, First Avenue at 16th Street, Suite 2S34H, New York, NY 1003 USA

**Keywords:** People who use drugs, Social ecological model, Coronavirus (COVID-19) pandemic, COVID-19

## Abstract

The COVID-19 pandemic has resulted in unparalleled societal disruption with wide ranging effects on individual liberties, the economy, and physical and mental health. While no social strata or population has been spared, the pandemic has posed unique and poorly characterized challenges for individuals with opioid use disorder (OUD). Given the pandemic’s broad effects, it is helpful to organize the risks posed to specific populations using theoretical models. These models can guide scientific inquiry, interventions, and public policy. Models also provide a visual image of the interplay of individual-, network-, community-, structural-, and pandemic-level factors that can lead to increased risks of infection and associated morbidity and mortality for individuals and populations. Such models are not unidirectional, in that actions of individuals, networks, communities and structural changes can also affect overall disease incidence and prevalence. In this commentary, we describe how the social ecological model (SEM) may be applied to describe the theoretical effects of the COVID-19 pandemic on individuals with opioid use disorder (OUD). This model can provide a necessary framework to systematically guide time-sensitive research and implementation of individual-, community-, and policy-level interventions to mitigate the impact of the COVID-19 pandemic on individuals with OUD.

## Background

The COVID-19 pandemic has disrupted all aspects of society, yet poses unique risks to individuals with opioid use disorder (OUD). To inform the rapidly evolving, time-sensitive efforts to understand and address the impact of severe acute respiratory syndrome coronavirus 2 (SARS-CoV-2) and the associated disease, COVID-19, on individuals with OUD, a conceptual model that comprehensively identifies and unifies the range of relevant factors is needed. Herein, we propose an adaption of the widely-cited social ecological model (SEM) that may serve as a foundation for ongoing and future work on addressing COVID effects among individuals with OUD [1].

### A modified version of the social ecological model

**Fig. 1:**
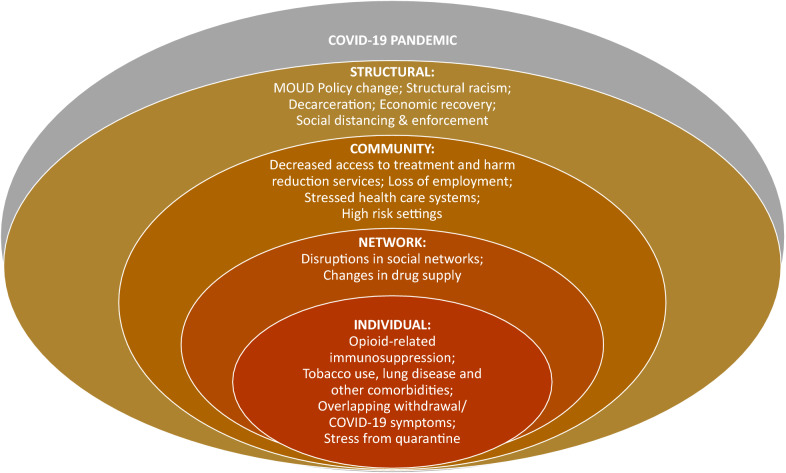
Social Ecological Model of Factor Impacting the Health of Individuals with Opioid Use Disorder from COVID-19. Figure Adapted from Baral et al. [1]

We describe how each of the layers of the SEM (individual, network, community, structural, and pandemic) interact to affect the health of individuals with OUD during the COVID-19 pandemic (Fig. 1). Factors can act at multiple layers and interact to influence the burden of COIVD-19 among individuals with OUD.

***Individual factors*** represent biological or behavioral characteristics associated with an individual’s risk of infection and associated morbidity and mortality.

*Individual behavioral factors*: Individuals with OUD may face elevated risk of exposure to SARS-CoV-2 due to increased risk of being in crowded conditions such as shelters, unstable housing conditions, or shared living spaces, in comparison to the general population. While other population subgroups, such as multigenerational families living in the same household may also be at increased risk of SARS-CoV-2 exposure, individuals with OUD make up a disproportionate share of the sheltered and unstably housed population clearly elevating their risk of exposure to SARS-CoV-2 [2]. While it can be difficult for individuals in the general population to maintain social distancing during activities such as food shopping or going to the pharmacy to pick-up medications, we would argue that these challenges are both smaller in magnitude and less frequent compared to many individuals with OUD. For example, patients receiving methadone from an opioid treatment program (OTP) must present daily for directly observed daily dosing. While some of these requirements have been loosened to allow for take home methadone doses, this option may not be available to all patients and methadone initiation still requires an in-person visit [3]. Daily clinic visits strain the ability to maintain social distancing and increase likelihood of potential exposure to SARS-CoV-2. For individuals with OUD not in treatment, there can be risks of exposure to SARS-Cov-2 on a daily or more frequent basis when they make contact with people to obtain illicit opioids (i.e., dealers). Furthermore, the need to address opioid use and sequelae by accessing harm reduction services and/or naloxone may be a challenge to social distancing, thereby increasing infection and transmission risk. As COVID-19 prevalence fluctuates, these behavioral characteristics become more dangerous for the individual and communities as they could increase the risk of disease acquisition and propagation.

*Individual biological factors*: Individuals with OUD may be at higher physiologic risk for infection and serious sequelae from SARS-Cov-2 due to opioid and other substance use-related immunosuppression and comorbidity, including concomitant structural lung disease [4]. There is also a concern that opioid-related respiratory depression may increase the risks of hypoxemia associated with COVID-19 viral pneumonia leading to worse clinical outcomes [4]. The aforementioned individual biological factors are likely playing a role in findings from a large case control study demonstrating that individuals with a recent OUD diagnosis had significantly higher risks of COVID-19 diagnosis, hospitalization, and death than those with either other substance use disorders or no substance use disorder [5].

***Network factors*** connect individuals to society and include interpersonal relationships, social networks, friends, and family. Among individuals with OUD, strong network cohesion can protect against risky opioid use and overdose while network dissolution may be a risk factor for opioid-related health consequences.

*Social networks*: A strong social network can be protective against the added stress associated with COVID-19 and adverse consequences among individuals with OUD by discouraging return to use and encouraging engagement in treatment. In contrast, diminishment of social network ties may lead to reduced treatment or harm reduction service engagement or to solitary drug use with absence of someone to mitigate opioid-related harms (e.g. naloxone administration in the setting of overdose). Disrupted social networks and social isolation as a result of pandemic-related stay at home orders may contribute to anxiety and depression, which may trigger increases and/or return to opioid use or multi-substance use increasing the risk of overdose.

*Drug networks*: Disruptions in drug markets and trafficking patterns due to the pandemic may also increase overdose risk and multi-substance use by leading to an increase in more harmful, domestically produced substances and increased use of pharmaceutical products that may be more readily available, such as benzodiazepines [6].

*Network disruptions* are important to measure and can include inquiries about drug-use networks, supply networks, family and social support. It is possible that individuals with limited ability to connect remotely will have more pronounced network disruptions. Literature suggests that individuals with OUD, especially in rural areas, lack the internet services needed to access telemedicine and video- based communication [7]. Such individuals could be at risk from more severe social isolation or by seeking out more face-to-face contact for social interactions or treatment acquisition, be at greater risk of COVID-19 acquisition. How individuals choose to use drugs within their network can have positive and negative implications for both COVID-19 acquisition and overdose. On the one hand, the sharing of drugs, which comprises an important component for many patients with OUD, increases the risk of COVID-19 acquisition but decreases the risk of fatal overdose by having someone nearby who can administer naloxone. On the other hand, solitary use increases overdose risk but decreases the risk of COVID-19 acquisition.

***Community factors*** describe relationships of networks and organizations that operate within defined boundaries [1]. Relevant factors are those that provide safe, evidence-based treatment and harm reduction services, housing, employment and medical care. Also pertinent are those related to incarceration, since individuals with OUD comprise a disproportionate share of the jail and prison population [2].

*Treatment and harm reduction communities* are especially significant to the current COVID-19 pandemic given the near complete disruption of these services in some locations. In other localities, treatment and harm reduction efforts have transformed service provision through increased use of telemedicine and improved distribution of personal protective equipment (PPE) to ensure safe street outreach. Despite new delivery models, a number of barriers to adequate service provision for individuals with OUD and the population more generally persist. These include continued lack of adequate PPE for clinic staff, inadequate COVID-testing services, limited internet service, lack of a phone and a private place for phone usage. These community disruptions and adaptations should be catalogued and measured.

*Other community-level factors* that could increase risk for individuals with OUD are greater exposure to high-risk settings such as homeless shelters, communal living, and incarceration all of which have increased risk of COVID-19 acquisition and transmission [8].

Lastly, the *economic collapse* associated with the COVID-19 pandemic will have sweeping effects on society as a whole but could be especially damaging to individuals with OUD who are more likely to be of low socioeconomic status and are often dependent on community-level safety nets. On one hand, individuals of low socioeconomic status are more likely to work in “essential” jobs such as food service, environmental service and retail, placing them at higher risk of COVID-19 exposure and the associated health risks and psychological stressors. On the other hand, economic stressors resulting from job loss are likely to act synergistically, in a negative way, with the psychological stressors brought on by social isolation. While these stressors are not unique to individuals with OUD, in this population, they could lead to an increased risk of both intentional and unintentional overdose. Financial stress to healthcare systems could also negatively affect individuals with OUD if safety net medical programs are curtailed due to lack of funding.

***Structural factors*** frame and constrain community level response. They are mediated through laws and policies as well as society and economics. When structural factors are implemented in a disproportionate or discriminatory fashion, they can have negative consequences on the most marginalized populations. Conversely, structural elements can be protective if they aim to improve equity and access to resources for high-risk populations. Concerning the COVID-19 pandemic, policy factors have been central to shaping community level response that impact individuals with OUD.

*Drug policy changes*: Specifically, there have been changes in Substance Abuse and Mental Health Services Administration policies loosening restrictions for medications for opioid use disorder (MOUD prescribing and distribution [3]. These policy changes increased flexibility to initiate and maintain OUD treatment while maintaining social distancing, thereby reducing exposure risk. In addition to the previously-mentioned barriers to implementing these policy changes, concern has been raised that loosening restrictions for take home methadone could lead to increased overdose risk if applied universally to all patients, regardless of clinical and psychological stability [9]. Conversely, loosening restrictions on methadone take-homes could also lead to increased rates of diversion possibly creating risk of harm to individuals who are not in methadone treatment.

*Decarceration*: Another significant structural change has been decarceration, which reduces prison overcrowding, and the unacceptably high transmission risk associated with high density, confined jail and prison settings [8]. Yet, decarceration must be coupled with additional structural approaches including increased financial support for re-integration services. Given that overdose risk is highest immediately following release from incarceration, a lack of re-entry planning that links individuals with OUD to outpatient MOUD treatment, could lead to increased overdose risk in this population.

*Social/physical distancing*: The most significant policy changes, however, have been those associated with social distancing. While such polices have affected all communities, they likely have disproportionate impacts on individuals with OUD through mediators like decreased access to harm reduction services, loss of employment, and disruptions in addiction treatment. Structural racism is likely to accentuate these disparities in minority communities and be particularly detrimental for those communities with high densities of individuals with OUD. Individuals with OUD were already highly stigmatized and marginalized before the pandemic. These characteristics made them more likely to be economically insecure and unstably housed.

*Other structural factors*: Structural changes and policies geared toward general economic recovery and housing stability will take time to reach individuals with OUD. Separately, there is concern that OUD-related stigma, lack of usual source of care including harm reduction, and potential consequences of a positive COVID test, such as loss of housing, could potentially drive individuals with OUD away from seeking testing even when clinically indicated. Lastly, increasing use of law enforcement to police the pandemic may contribute to risk by driving substance use underground, further inhibiting treatment seeking, and preventing individual who use drugs from interacting to save lives by administering naloxone due to worry about police intimidation, arrest, and incarceration.

***Stage of the epidemic*** is what determines the risk in each model strata. As the COVID-19 infection risk decreases, acquisition risk and associated morbidity and mortality for individuals with OUD within each layer decreases, but does not disappear. Individual factors that increase susceptibility to COVID-19 infection and disease related morbidity and mortality are still at work and may not be easily modifiable. The biologic and social-behavioral risks of infection intrinsic to individuals OUD means that an uptick in COVID-19 infections is always possible. Increasing incidence of COVID-19 in individuals with OUD could drive progression of the epidemic into networks and communities. Resulting structural changes put in place to control disease spread could result in increasingly oppressive policy measures that could further degrade networks and communities increasing both OUD and COVID-19 disease risk.

### Using the model in practice

Used in practice, the proposed model can improve COVID-19 prevention and treatment research studies among individuals with OUD. First, the model can inform the development of observational studies that seek to identify factors associated with COVID-19 acquisition and disease severity in this population, as well as influences of the pandemic on initiation of opioid use, interruptions in treatment for opioid use disorder, and overdose risk. Primary data collection studies should develop questionnaires and electronic health record-based studies should assess individual-level (i.e. comorbidity burden, overdose), network-level (i.e. social isolation and how patients are navigating complex network issues), and structural factors (i.e., community-level access to treatment, resources during the pandemic) driving risk and severity of infection. For example, mapping temporal changes in network size and composition and correlations with solitary drug use and overdose over the course of the COVID-19 pandemic is critical for understanding COVID-related overdose risk. The model also can inform development of randomized trials to reduce COVID-19 effects among individuals with OUD. Pre-exposure prophylaxis, treatment, or vaccine trials should aim to recruit diverse study populations including individuals with OUD. Given efficacy of COVID-19 treatment and prevention may vary by a range of attributes and hence differ among individuals with OUD versus those who are not, subgroup analyses should accompany intention to treat analyses to best understand uptake, adherence, and efficacy among individuals with OUD. In addition, we need observational studies and trials that test programs implemented in the context of the COVID-19 pandemic that aim to improve access to treatment, naloxone, and harm reduction for individuals with OUD. Data from evaluations of novel programs implemented in settings hit hard and early by COVID-19 should inform how the next US region that is impacted by COVID-19 can respond to best assure protection of individuals with OUD against overdose, COVID-19, and social, economic, and health harms.

### Continuing model relevance

We believe our conceptual model will retain significance even after a vaccine becomes available. Clusters continue to emerge and re-emerge in the US and globally, disproportionately infecting our most vulnerable groups including those with OUD, who experience disproportionate risk. The inevitable vaccine shortage will likely lead to delays in vaccination in highly marginalized members of the general population including those with OUD who tend to have limited access and exposure to the healthcare system. Adding concern is the fact that when vaccination becomes available to those with OUD, uptake may be low as prior studies have demonstrate that rates of vaccination for other viral diseases, such as hepatitis A and B are low in patients with OUD [10]. Given the aforementioned factors, the COVID-19 pandemic is likely to be a considerable public health issue in patients with OUD for the foreseeable future.

## Conclusions

As the COVID-19 pandemic continues, rapid generation of research and its impact on various populations will be crucial. Our proposed modified social ecological model provides a comprehensive approach for conceptualizing the impact of the current COVID-19 context on individuals with OUD.

## Data Availability

Not applicable.

## Abbreviations

OUD: Opioid use disorder
SARS-CoV-2: Severe acute respiratory syndrome coronavirus 2
OTP: Opioid treatment program
MOUD: Medications for opioid use disorder
